# Tracking Water, Sanitation, and Hygiene Practices: Waste Management and Environmental Cleaning in the Slums of North India

**DOI:** 10.7759/cureus.42067

**Published:** 2023-07-18

**Authors:** Abhishek Gupta, Mili Sengar, Manish Manar, Utkarsh Bansal, Shivendra K Singh

**Affiliations:** 1 Community Medicine, Government Medical College, Kannauj, Kannauj, IND; 2 Community Medicine, T.S. Misra Medical College, Lucknow, IND; 3 Community Medicine, King George’s Medical University, Lucknow, IND; 4 Pediatrics, Hind Institute of Medical Sciences, Barabanki, IND

**Keywords:** handwashing, toilet, disposal, defecation, pakka, kaccha, slum

## Abstract

Background

One or more of the following five amenities is lacking in slum communities: durable housing, sufficient living area, access to clean water, access to improved sanitation facilities, and secure tenure. This study aimed to identify the gaps in water, sanitation, and hygiene conditions in the urban slums of Lucknow.

Methodology

A community-based, cross-sectional study was conducted among families residing in the urban slums of Lucknow, Uttar Pradesh, India for 18 months starting from April 2020.

Results

A total of 747 heads of families were interviewed and their families were surveyed. The proportion of kaccha slums was 37.25% and of pakka slums was 62.74%. About 98.3% of families residing in kaccha slums used indiscriminate throwing as a method of solid waste disposal. About 96.5% of families residing in kaccha slums practiced open-field defecation while those residing in pakka slums used a toilet within the premises. Kaccha slum dwellers were practicing open-field defecation 12.8 times more than pakka slum dwellers. This study showed that sanitary conditions in kaccha slums were mainly responsible for the overall burden of excreta disposal, solid waste disposal, and access to water supply for drinking and other household purposes.

Conclusions

Water supply and housing conditions such as dampness, floor, and the non-availability of electricity are the primary predictors of the preference for open-field defecation among slum dwellers.

## Introduction

The United Nations defines slums as communities lacking in one or more of the following five amenities: durable housing, sufficient living area, access to clean water, access to improved sanitation facilities, and secure tenure [1]. The proportion of the urban population living in slums worldwide grew to 23.5% in 2018, and the absolute number of people living in slums or informal settlements grew to over 1 billion [2].

In 1981, the slum population in India constituted 17.5% of the urban population which increased to 35% in 2018 [3]. Slums are very common in India and are present in around 65% of Indian towns. Despite this, slum lives are the most overlooked section of society. Six out of 10 slum dwellers live close to unsanitary drains, and almost four of every 10 do not have access to treated water [4].

Rapid urbanization has resulted in the migration of people from rural areas to cities. According to the Central Statistical Organization, the slum population of Uttar Pradesh was 58.4 lacs in 1991, 77.10 lacs in 2001, and 1.02 crore in 2011, which is 23.05% of the urban population according to the 2011 Census [5].

By 2050, the urban population of India will be almost double of 2014 [6]. Access to water, sanitation, and hygiene (WASH) is a fundamental human right [7]. The quality of WASH services has been unable to keep pace with the growing population. Much of the population living in urban slums is deprived of their basic right to clean drinking water and sanitation. This, in turn, has serious adverse effects on the growth and development of children and their overall survival. Good hygiene practices are necessary and critical for the prevention of the spread of infectious diseases as well as for growth and survival. It also prevents children from missing school which ultimately leads to better a future for them and for the country [8].

Currently, nearly 15% of people still practice open defecation in India [9]. In India, according to the National Family Health Survey-5 (2019-2020), 5% of households do not have access to an improved source of drinking water, and almost 30% of households do not use an improved sanitation facility [10].

The Sustainable Development Goals aim to achieve universal access to WASH. The government has accelerated the focus on sanitation and universal sanitation coverage through the Swachh Bharat Mission (SBM) launched on October 2, 2014, by the Prime Minister of India.

The Swachh Bharat Mission (Urban) (SBM-U) had three major objectives, namely, achieving 100% open defecation-free (ODF) status, solid waste management (SWM), and behavior change through Jan Andolan, by October 2, 2014, in all statutory towns. Under SBM-U, urban India has achieved ODF status, a fitting tribute to Mahatma Gandhi’s vision. The sanitation objective of the mission has been fulfilled, and lakhs of citizens, predominantly women, have been provided dignity and safety. Additionally, there has been a marked reduction in vector-borne diseases along with improvement in health indicators, setting urban India on the path of holistic cleanliness. The National Sample Survey Office had undertaken an impartial assessment of the mission in 2018. In its report at the 76th Round (with the theme of Drinking Water, Sanitation, Hygiene and Housing Conditions of India: July-December 2018), the study found that 98% of toilets are being used in urban areas. The mission has been extended for five years, i.e., from October 1, 2021, to October 1, 2026, as SBM-U 2.0, for completing the remaining work, with a re-emphasis on swachh (clean) behavior and sustaining it [11].

This study aims to identify the gaps in access to WASH in the urban slums of Lucknow and suggest appropriate future interventions.

## Materials and methods

Study sample

A community-based, cross-sectional study was conducted among families residing in the urban slums of Lucknow, Uttar Pradesh. Lucknow is the capital of Uttar Pradesh state in India and is located 123 m above sea level. The total population of Lucknow is 2,817,105, 12.95% of which reside in slums with substandard living conditions [12]. The study was conducted for 18 months starting from April 2020 (the survey duration was stretched because of the COVID-19 lockdown in 2020).

Sampling technique

Urban Lucknow is divided into six zones by Lucknow Municipal Corporation. Of the six zones, one zone (zone 5) was randomly selected. There are 609 notified/recognized slums in urban Lucknow [13]. In the selected zone, there were 123 slums, of which six were selected randomly by simple random sampling. Among these six slums, three were kaccha slums and three were pakka slums. A total of 801 families were residing in these selected slums, and all of them were included in the study. The purpose of the study was explained in the local language and consent for participation was obtained from each of the study participants. After applying inclusion and exclusion criteria and obtaining verbal consent from the heads of families, 747 heads of families were interviewed, and their housing and sanitary conditions were assessed.

We included families residing in the slum area for more than six months who were able to converse in Hindi and/or English language. We excluded family members who could not be located after three attempts.

Operational definitions

Slum

(1) All specified areas in a town or city notified as a Slum by the State/Local Government and UT Administration under any act including the Slum Act [12]. (2) All areas recognized as a Slum by the State/Local Government and UT Administration, Housing and Slum Boards, which may have not been formally notified as a slum under any act. (3) A compact area of a population of at least 300/about 60-70 households of poorly built congested tenements in unhygienic environments usually within adequate infrastructure and lacking proper sanitary and drinking water facilities.

*Kaccha and Pakka Slum*s

Kuccha slums were slums containing kaccha houses. Kaccha houses refer to walls and roofs made of materials other than concrete (e.g., corrugated galvanized sheets) [14]. Pakka slums were slums containing pakka houses. Pakka houses refer to houses with roofs made of concrete [15].

Data collection

The data was collected using a predesigned, pretested, and semi-structured questionnaire. Information was collected on demography, socioeconomic status, cooking fuel, and WASH practices. The Joint Monitoring Program 2017 definitions were used for improved sources of drinking water and sanitation [16].

Ethical clearance

Ethical clearance was obtained from the Institutional Human Ethical Committee of the Institute of Hind Institute of Medical Sciences (IEC/IRB number: HIMS/IRB/2019-20/1G.0'A-/l) before initiating the study. The purpose of the study was explained in the local language and consent for participation was obtained from each of the study participants.

Study variables

The following variables were studied: dependent variables including excreta disposal and solid waste disposal; independent variables including age, sex, marital status, education, occupation, and housing conditions such as locality, house, dampness, floor, wall, roof, electricity, water supply, drinking water, hand washing, and washing uncooked food before eating.

Data analysis

SPSS statistical software version 21.0 for Windows (IBM Corp., Armonk, NY USA) was used for data analysis. Descriptive statistics such as frequency and percentage for categorical variables were determined. The chi-square test was used to assess the relationship between independent and dependent variables. Two-tailed probability was calculated to test statistical significance at the 5% level of significance.

## Results

Of the total surveyed slum population, the majority (84.5%) were Hindus. Among kaccha slums, Muslims accounted for 38.7% of the total population. Regarding the age group, the majority (48.2%) were in the 20-45-year age group. Among the kaccha slum population, 50.2% were illiterate, while this percentage was 13% in the pakka slum. In the kaccha slum, 44.3% were daily wage workers, had no fixed job, or they did not want to share about their occupation, while in pakka slum, it was 14.7% (Table 1).

**Table 1:** Biosocial profile of the surveyed slum population. *: Children up to seven years of age who were not enrolled in any formal or non-formal education system; +: Students of any age/age up to 14 years who were not studying; ++: Daily wage worker/No fix job/Do not want to share

Biosocial variables		Kaccha slum, n (%)	Pakka slum, n (%)	Total, n (%)
Religion/Nationality	Hindu	763 (61.3)	2,060 (98.2)	2,823 (84.5)
	Muslim	482 (38.7)	19 (0.9)	501 (15.0)
	Christian	0	12 (0.6)	12 (0.3)
	Bengali	0	6 (0.3)	6 (0.2)
Sex	Male	630 (50.6)	1,077 (51.4)	1,707 (51.1)
	Female	615 (49.4)	1,020 (48.6)	1,635 (48.9)
Age group (year)	Up to 5	131 (10.5)	172 (8.2)	303 (9.1)
	5–10	110 (8.8)	133 (6.3)	243 (7.3)
	10–20	299 (24.0)	383 (18.3)	682 (20.4)
	20–45	577 (46.3)	1,033 (49.3)	1,610 (48.2)
	45–60	108 (8.6)	231 (11.0)	339 (10.1)
	≥60	20 (1.6)	145 (6.9)	165 (4.9)
Education	Illiterate	625 (50.2)	272 (13.0)	897 (26.8)
	Class 6th/Non-formal education	379 (30.4)	448 (21.4)	827 (24.7)
	7th–12th class	100 (8.0)	727 (34.7)	827 (24.7)
	Graduate and above	19 (1.5)	542 (25.8)	561 (16.8)
	Not applicable^*^	122 (9.9)	108 (5.1)	230 (6.90)
Marital status	Married	610 (49.0)	997 (47.5)	1,607 (48.1)
	Unmarried (age 21 years and above)	60 (4.8)	268 (12.8)	328 (9.8)
	Unmarried (age below 21 years)	556 (44.7)	740 (35.3)	1,296 (38.8)
	Widow, widower, divorced, separated	19 (1.5)	92 (4.4)	111 (3.3)
Occupation	Government job/Pensioner	04 (0.3)	110 (5.2)	114 (3.4)
	Private job	68 (5.5)	299 (14.3)	367 (11.0)
	Business	30 (2.4)	96 (4.6)	126 (3.8)
	Homemaker	195 (15.7)	504 (24.0)	699 (20.9)
	Student/Not applicable^+^	397 (31.9)	779 (37.1)	1,176 (35.2)
	Other^++^	551 (44.3)	309 (14.7)	860 (25.7)
Total		1,245 (37.25)	2,097 (62.75)	3,342 (100)

Table 2 shows that 33.3% of slum families were living in congested localities. Most of the kaccha slum families were residing in rented houses (96.2%). While the majority (83.6%) of pakka slum families were residing in their own houses. Dampness was present in 73.1% of slum households. Electricity was present in only 52.9% of kaccha slum households.

**Table 2:** Environmental conditions of the surveyed slums (housing and waste disposal).

Environmental condition		Kaccha slum, n (%)	Pakka slum, n (%)	Total, n (%)
Locality (p = 0.04)	Congested	449 (36.0)	695 (33.1)	1,144 (34.2)
	Semi-congested	744 (59.8)	1,336 (63.7)	2,080 (62.3)
	Open	52 (4.2)	66 (3.2)	118 (3.5)
House (p < 0.001)	Rented	1,192 (95.7)	284 (13.5)	1,476 (44.2)
	Own	53 (4.3)	1,813 (86.5)	1,866 (55.8)
Dampness (p < 0.001)	Yes	1,005 (80.7)	1,405 (67.0)	2,410 (72.1)
	No	240 (19.3)	692 (33.0)	932 (27.9)
Floor (p < 0.001)	Kaccha	1,181 (94.9)	51 (2.4)	1,232 (36.9)
	Pukka	64 (5.1)	2,046 (97.6)	2,110 (63.1)
Wall (p < 0.001))	Brick without cement	996 (80.0)	0 (0.0)	996 (30.0)
	Brick without plaster	207 (16.6)	154 (7.3)	361 (10.7)
	Brick with plaster	42 (3.4)	1,943 (92.7)	1,985 (59.3)
Roof (p < 0.001)	Thatched/Tiles	54 (4.3)	41 (2.0)	95 (2.8)
	Sheet	1,144 (91.9)	92 (4.3)	1,236 (37.0)
	Concrete/Brick	47 (3.7)	1,964 (93.7)	2,011 (60.2)
Electricity (p < 0.001)	Yes	637 (51.2)	2,080 (99.2)	2,717 (81.3)
	No	608 (48.8)	17 (0.8)	625 (18.7)
Total		1,245 (37.25)	2,097 (62.75)	3,342 (100)

Table 3 shows that around two-thirds (75.4%) of kaccha slum dwellers were dependent on privately paid water supply for their daily household needs, while 16.3% were dependent on government water supply (outside the premises), and 6.9% were dependent on a hand pump/jet pump situated outside the premises. About 98.3% of kaccha slum families used indiscriminate throwing as a method of solid waste disposal. About 96.5% of kaccha Slum families preferred open-field defecation, while pakka slum families used a toilet within the premises (99.8%).

**Table 3:** Water, hygiene, and sanitation practices.

Water, hygiene, and sanitation practices		Kaccha slum, n (%)	Pakka slum, n (%)	Total, n (%)
Water supply (p < 0.001)	Government water supply	17 (1.4)	2,068 (98.6)	2,085 (62.4)
	Hand pump/Jet pump (outside the premises)	78 (6.3)	5 (0.2)	83 (2.5)
	Privately paid water supply	934 (75)	5 (0.2)	939 (28.1)
	Government supply with an additional source	0 (0)	19 (0.9)	19 (0.6)
	Government water supply (outside the premises)	216 (17.3)	0 (0)	216 (6.4)
Drinking water	Continuous	100 (8.1)	200 (9.5)	300 (9.0)
	Intermittent	1,145 (91.9)	1,897 (90.5)	3,042 (91.0)
Water treatment before drinking	Boiling	0 (0)	15 (0.7)	15 (0.45)
	Chlorine tablets	0 (0)	0 (0)	0 (0)
	Cloth to strain	0 (0)	10 (0.5)	10 (0.3)
	Filters	0 (0)	2,072 (98.8)	2,072 (62)
	None of the above	1,245 (100)		1,245 (37.25)
Hand washing	Before every meal	998 (80.2)	2,097 (100)	3,097 (92.7)
	After using toilet	956 (76.8)	2,097 (100)	3,053 (91.4)
Food eating from street vendor	Daily	45 (3.6)	268 (12.8)	313 (9.4)
	Once a week	197 (15.8)	298 (14.2)	485 (14.5)
	More than once a week	986 (79.2)	1,467 (70.0)	2,443 (73.1)
Washing uncooked food before eating	Yes	56 (4.5)	2,087 (99.5)	2,132 (63.8)
	No	1,189 (95.5)	10 (0.48)	1,199 (35.9)
Solid waste disposal (p < 0.001)	Indiscriminate	1,223 (98.2)	50 (2.4)	1,273 (38.1)
	Nagar Nigam	22 (1.8)	2,029 (96.8)	2,051 (61.4)
	Nagar Nigam + indiscriminate	0 (0)	18 (0.9)	18 (0.5)
Excreta disposal (p < 0.001)	Open-field defecation	1,201 (96.5)	5 (0.2)	1,206 (36.1)
	Toilet within the premises	44 (3.5)	2,092 (99.8)	2,136 (63.9)
Total		1,245 (37.25)	2,097 (62.75)	3,342 (100)

Table 4 shows that the residents of kaccha slums were practicing open-field defecation 12.8 times more than those residing in pakka slums. Similarly, residents of houses without electricity preferred open-field defecation (7.59 times). Residents with water sources outside the premises also preferred open-field defecation in comparison to those who had government water supply within the premises (17.9 to 669.9 times).

**Table 4:** Logistic regression analysis for excreta disposal among the study population.

Variable		Univariate		Multivariate	
		Exp (B)	95% CI	Exp (B)	95% CI
Religion	Hindu	Ref			
	Muslim	46.3	31.54–68.02	--	
	Other	p = 0.99			
Sex	Male	Ref			
	Female	1.04	0.90–1.2	--	
Age group (year)	15–64	Ref			
	<15	1.68	1.44–1.97	--	
	>64	0.50	0.07–0.35	--	
Education	Not applicable/Below 7 years	14.2	10.21–19.75	--	
	Illiterate	28.3	22.15–36.39	--	
	Class 6th/Non-formal education	10.6	8.32–13.61	--	
	7th class and above	Ref			
Marital status	Married	Ref			
	Unmarried (age 21years and above)	0.36	0.26–0.49	--	
	Unmarried (age below 21 years)	1.22	1.05–1.42	--	
	Widow, widower, divorced, separated	0.35	0.21–0.58	--	
Occupation	Fixed job/Business	Ref			
	Homemaker	1.99	1.48–2.57	--	
	Student/Not applicable	2.56	1.99–3.28	--	
	Daily wages/No fix job	8.98	6.93–11.62	--	
Slum	Kaccha	11,420.4	4,516.33–28,878.73	12.8	1.6–96.9
	Pakka	Ref			
House	Rented	239.9	163.6–351.6	--	
	Owned	Ref			
Dampness	Yes	2.27	1.91–2.70	7.59	1.93–29.7
	No	Ref			
Floor	Kaccha	1,535.6	973.85–2,421.47	10.05	2.49–40.69
	Pukka	Ref			
Electricity	Yes	Ref			
	No	55.68	39.3–78.88	7.79	1.44–42.31
Water supply	Government water supply	Ref			
	Hand pump/Jet pump (outside the premises)	824.6	306.8–2215.9	17.9	2.1–153.5
	Privately paid water supply	43,379.3	14,498.4–129,790.5	669.9	71.8–6,249.9
	Government water supply (outside the premises)	p = 0.99		--	

## Discussion

A report published in 2015 that aimed to analyze the slum situation in Lucknow [17] reported that a significant proportion of the minority population was living in slums. About 19% of the slum population belonged to the minority group, and individuals aged >65 years constituted 6% of the slum population. In this study, in kaccha slum 38.7% and in pakka slums 1.8% of the population belonged to the minority group. Among the age group of 60 years or more, the population in kaccha and pakka slums was 1.6% and 6.9%, respectively. Nearly 37% of the slum population did not have access to a dependable occupation and secure income, whereas it was 44.3% in kaccha slums and 14.7% in pakka slums in this study. A significant proportion (73%) of the slum households did not have their own water supply connection and they depended on public taps, hand pumps, and neighboring households, whereas in this study, the proportion was 98.6% in kaccha slums and 0.4% in pakka slum. About 27% of slum households were practicing open defecation, whereas it was 96.5% in kaccha slums and only 0.2% among pakka slum residents in this study. In about 32% of the slums, the collection of waste was entirely non-existent, whereas in the present study, in pakka slums it was 2.4% and in kaccha slums it was 98.2%.

In the above-mentioned report [17], the proportion of kaccha slums was 7.9% and that of pakka slum/semi-pakka slums was around 92%, while in this study the proportion of kaccha slums was 37.25% and of pakka slums was 62.75%. This difference may be due to different sampling techniques. Another reason for the difference in observations can be because the study [18] used data from the Rajiv Awas Yojana primary survey of 2011, after which marked improvement occurred in slum infrastructure. Because this study started in December 2019 (after the implementation of Rajiv Awas Yojna), the results were better.

In a study conducted in the slums of Vellore, the majority of the study participants belonged to the age group of 21-30 years (27%) and were Hindus (88%). The majority of them were either uneducated or had attended up to middle school (29% each). More than half of the study population was engaged in some form of unskilled work (54%) and lived in a pucca dwellings (52%) [19]. These findings were similar to those of the present study. It was found that only one slum had a public toilet facility, but it was not used by the locals. Open defecation was practiced by people from all four slums. Three out of four slums used drains as waste disposal areas, and flood areas were present in all four slums. Out of the total, 62.3% of respondents depended on public taps as the major source of drinking water, 31.5% purified water before drinking, 68.5% allowed water to stand for a day before use, 86.5% practiced hand washing before meals, 33.2% practiced open defecation, 7% and 74% used soap and water for hand washing before meals and after defecation, respectively, 68.3% threw liquid wastes haphazardly, and 59.8% threw solid wastes haphazardly into drains [19].

A community-based, cross-sectional study was conducted in the urban slums of Lucknow city in 2014 among a total of 384 households who were assessed for housing and sanitary conditions. Among 384 households included in the study, 77.1% were situated in congested localities. A total of 74.5% of the houses had unsafe practices for water storage and handling. The practice of hand washing before eating was found in 52.6%. Similar results were found in this study. A total of 51.1% of the households reported open-field defecation, which is higher than this study, indicating that the initiative of Swachh Bharat Abhiyan has led to improvement since 2014 [20].

Findings similar to this study were reported by Kanungo et al. in prospective enteric fever surveillance conducted in two municipal wards of Kolkata and Information on WASH practices which was collected in two surveys (2018 and 2019). Over 90% of households had access to piped water, and 6% reported access to continuous supply. Access to improved latrines was almost universal, although 80% used shared facilities. Unhealthy disposal of children’s stools was reported in both rounds. Food hygiene practices were high, with most (>90%) washing uncooked items before eating. Additionally, it was noted that street foods were consumed frequently [21]. These findings were similar to the present study.

A cross-sectional study was performed by Undavalli et al. [22] in the field practice area of a private medical college in Andhra Pradesh from January to March 2014. A total of 200 households constituted the sample, and it was found that 100% of households had access to improved drinking water sources, but improved sanitation facilities were observed among 58%, which is lower than the present study. This can be attributed to the fact that the study was conducted in 2014 and much improvement has occurred since then.

Study limitations

The population of slums keeps changing from time to time due to the high urbanization and migration rates (the slum population keeps moving in search of better job opportunities and livelihood); hence, the background characteristics of the study subjects may have changed in due course of time.

## Conclusions

The present study showed that sanitary conditions in kaccha slums were very poor. Kaccha slums were mainly responsible for the overall burden of excreta disposal, solid waste disposal, and access to their own water supply for drinking and other household purposes. Regression analysis in this study also showed that water supply and housing conditions such as dampness, floor, and nonavailability of electricity are primary predictors for open-field defecation as a choice of excreta disposal among slum dwellers. The study has reported low coverage of WASH. This study emphasizes the need for better WASH practices in urban slums and the requirement of better facilities for waste and excreta disposal.
